# Silencing of FABP1 ameliorates hepatic steatosis, inflammation, and oxidative stress in mice with nonalcoholic fatty liver disease

**DOI:** 10.1002/2211-5463.12240

**Published:** 2017-06-05

**Authors:** Takako Mukai, Miki Egawa, Tamaki Takeuchi, Hitoshi Yamashita, Tatsuya Kusudo

**Affiliations:** ^1^ Faculty of Human SciencesTezukayama Gakuin UniversitySakaiJapan; ^2^ Department of Biomedical SciencesCollege of Life and Health SciencesChubu UniversityKasugaiJapan

**Keywords:** FABP1, NAFLD

## Abstract

Nonalcoholic fatty liver disease (NAFLD) is increasing in prevalence worldwide and has been identified as a risk factor for cirrhosis and hepatocellular carcinoma. However, there is no effective pharmacologic treatment for NAFLD. FABP1 is a liver‐specific fatty acid‐binding protein (FABP) that plays important roles in intracellular lipid metabolism in the liver. We investigated the effect of repression of FABP1 expression on NAFLD, using adenovirus‐mediated silencing of FABP1. FABP1 knockdown in the liver decreased the liver weight and hepatic triglyceride (TG) accumulation. The expression of inflammatory and oxidative stress markers in the liver was also reduced. The level of thiobarbituric acid‐reactive substances, a marker of lipid peroxidation, in the liver of FABP1 knockdown mice was significantly decreased. These results suggest that FABP1 reduction in the liver is an effective approach against NAFLD.

**Abbreviations**
ER: endoplasmic reticulum
FABP: fatty acid‐binding protein
FFAs: free fatty acids
NAFLD: nonalcoholic fatty liver disease
NASH: nonalcoholic steatohepatitis
shRNA: short hairpin RNA
TBARS: thiobarbituric acid‐reactive substances
TG: triglyceride

Nonalcoholic fatty liver disease (NAFLD) is characterized by hepatic lipid accumulation without chronic excess alcohol intake and includes a spectrum of diseases from simple steatosis to nonalcoholic steatohepatitis (NASH) 1, 2. The prevalence of NAFLD is increasing and is now a public health problem worldwide. Approximately 10–20% of NAFLD patients develop NASH, which can progress to more serious diseases such as hepatic cirrhosis and hepatic cancer 3, 4. Therefore, the need for effective therapy in patients with NAFLD or NASH has increased. A number of studies have been carried out in order to understand the pathogenesis, diagnosis, and treatment of NAFLD. However, at present, there is no recommended treatment for the standard care of NAFLD patients. Currently, lifestyle interventions (diet and exercise) remain the standard treatment for patients with NAFLD/NASH 5. Although the detailed mechanisms underlying the progression from NAFLD to NASH are not yet fully understood, the fundamental cause is excessive hepatic lipid accumulation followed by lipotoxicity and/or oxidative stress 6, 7.

Fatty acid‐binding proteins (FABPs) are small cytoplasmic proteins that bind many hydrophobic ligands such as fatty acids 8, 9. FABPs are involved in intracellular lipid metabolism such as fatty acid uptake, transport, oxidation, lipid synthesis, and storage, and they play roles in nuclear receptor regulation 8. FABP1 is mainly expressed in the liver at very high levels (2–5% of cytosolic protein) and contributes to many biological processes in this tissue 10. Two laboratories have independently generated FABP1 gene knockout mouse lines on the C57BL/6 background 11, 12. The physiological importance of FABP1 in the regulation of lipid metabolism has been investigated using these mice 13, 14. The studies using these two FABP1‐deficient mouse lines suggest that FABP1 functions in hepatic fatty acid uptake, fatty acid oxidation, and very low‐density lipoprotein (VLDL) secretion 13, 14. Although there are contradictions in the results of the phenotypes between these two lines depending on diet, FABP1‐null mice were protected from developing high‐fat diet (HFD)‐induced triglyceride (TG) accumulation in the liver 15, 16. Thus, it is expected that pharmacological agents which attenuate FABP1 expression or function may suppress TG accumulation in the liver and ameliorate NAFLD. Although FABP1 is abundant in liver, it is also expressed in other tissues such as the small intestine and pancreas in rodents (also expressed in the kidney in human) 10, 17, 18. Therefore, the results obtained from experiments using global FABP1‐null mice are not specific for liver 19, 20. Moreover, these knockout mice are congenitally deficient in FABP1 expression, and thus they are not suitable for examining the therapeutic effect of FABP1 attenuation on NAFLD.

In this study, we used adenovirus‐mediated knockdown of FABP1 and investigated the effect of hepatic FABP1 repression on NAFLD.

## Materials and methods

### Animals

C57BL/6J male mice were obtained from Japan SLC (Hamamatsu, Japan). The mice were maintained at 23 ± 1 °C under artificial light for 12 h·day^−1^ and provided a standard chow diet (Diet No. CE‐2, 344 kcal/100 g, 11.6% kcal from fat; Clea Japan, Inc., Tokyo, Japan) and tap water *ad libitum*. C57BL/6J male mice were fed a HFD (60 kcal %‐fat, D12492; Research Diets, New Brunswick, NJ, USA) from the age of 3 months for 4 weeks. Then, the mice were divided into two groups and treated with Ad‐shLacZ or Ad‐shFABP1 described below. After virus infection, the mice were fed with HFD *ad libitum* for 1 week. All animal experiments were performed at Chubu University in accordance with the institutional guidelines for the care and use of research animals.

### Adenovirus preparation and *in vivo* animal injection

The DNA sequences corresponding to the short hairpin RNA (shRNA) sequences of FABP1: 5′‐CACCGAACTCAATGGAGACACAATCCGAAGATTGTGTCTCCATTGAGTTC‐3′ and 5′‐AAAAGAACTCAATGGAGACACAATCTTCGGATTGTGTCTCCATTGAGTTC‐3′ were annealed and ligated into the shRNA expression vector pENTR/U6 (Life Technologies, Carlsbad, CA, USA). Recombinant adenoviruses were generated according to the manufacturer's instructions. As a negative control, a recombinant adenovirus expressing a shRNA of LacZ was also generated. Adenovirus was purified by CsCl gradient centrifugation. The virus titer was determined by TCID50. Animals were anesthetized with 2–3% isoflurane in air and 0.2 mg of polyinosinic acid was injected via the orbital plexus 5 min prior to adenovirus injection. Recombinant adenovirus vectors (Ad‐shFABP1 and Ad‐shLacZ) were injected intravenously in 100 μL of saline at a dose of 4 × 10^8^ pfu. After 1 week, mice were killed and both tissue and blood samples were collected for storage at −80 °C or fixed in 4% paraformaldehyde/PBS for histological analysis.

### Lipid analysis

The serum levels of TG and cholesterol were determined using TG *E*‐Test Wako (Wako Pure Chemicals Industries, Ltd., Osaka, Japan) and Cholesterol *E*‐Test Wako (Wako Pure Chemicals Industries Ltd.), respectively. Liver lipids were extracted as described previously 21. Briefly, liver samples (30 mg) were homogenized in an extraction solution [hexane‐isopropyl alcohol (3 : 2, vol/vol)] using a Polytron tissue grinder. After centrifugation, the resultant supernatant was evaporated under reduced pressure. Samples were resuspended in 10% Triton X‐100 in isopropyl alcohol. TG and cholesterol were measured using TG *E*‐Test Wako and Cholesterol *E*‐Test Wako, respectively.

### TBARS assay

Thiobarbituric acid‐reactive substance (TBARS) levels were measured in liver homogenate using a TBARS Assay kit (Cayman Chemical, Ann Arbor, MI, USA) according to the manufacturer's protocol.

### Histological analysis

Oil Red O staining was performed as follows: Fixed tissues were embedded in O.C.T. compound and frozen. The frozen blocks were cut into 8‐μm thick sections and stained with Oil Red O. The sections were also stained with hematoxylin and eosin (H&E).

### Western blotting

Fifty milligrams of liver samples was homogenized in RIPA buffer (50 mm Tris‐HCl, pH 7.5, 150 mm NaCl, 1 mm EDTA, 0.1% SDS, 1% NP‐40, 0.5% sodium deoxycholate) containing protease inhibitor cocktail (Nakalai Tesque, Kyoto, Japan). The homogenate was then centrifuged at 12 000 ***g*** at 4 °C for 5 min and the supernatant was collected. Thirty micrograms of protein samples was separated by 12.5% SDS/PAGE and transferred onto a polyvinylidene difluoride (PVDF) membrane (Millipore, Bedford, MA, USA). The membrane was blocked with 5% nonfat dried milk in TBS containing 0.1% Tween‐20 (TBS‐T) for 1 h at room temperature and then incubated with primary antibody against FABP1 (Cell Signaling Technology, Danvers, MA, USA), β‐tubulin (Wako Pure Chemicals Industries Ltd.), and acetyl‐CoA carboxylase (ACC) (Cell Signaling Technology) overnight at 4 °C. The blots were then washed in TBS‐T three times and incubated with HRP‐labeled secondary antibody for 1 h at room temperature. After washing in TBS‐T three times, chemiluminescent signals were detected using immobilon‐P (Millipore, Bedford, MA, USA) and visualized with a light capture system (ATTO, Tokyo, Japan). The resulting images were quantified with Image J software (National Institutes of Health, Bethesda, MD, USA).

### Quantitative real‐time RT‐PCR

Total RNA was extracted using TRI Reagent (Molecular Research Center Inc., Cincinnati, OH) according to the manufacturer's protocol. Total RNA was reverse transcribed using high‐capacity cDNA reverse transcription kits (Applied Biosystems, Foster City, CA, USA), in accordance with the manufacturer's protocol. To quantify mRNA expression levels, real‐time RT‐PCR analysis was performed using a StepOne real‐time PCR system (Applied Biosystems) and PowerUp SYBR Green Master Mix (Thermo Fisher Scientific, Waltham, MA, USA). All gene expression data were normalized to 36B4. The oligonucleotide primer sets used are listed in supporting information (Table S1).

### Statistical analysis

Data were expressed as means ± standard error of the mean (SEM). Significant differences between groups were assessed by Student's *t*‐test.

## Results

### Adenovirus‐mediated FABP1 silencing reduced the expression of FABP1 in liver

**Figure 1:**
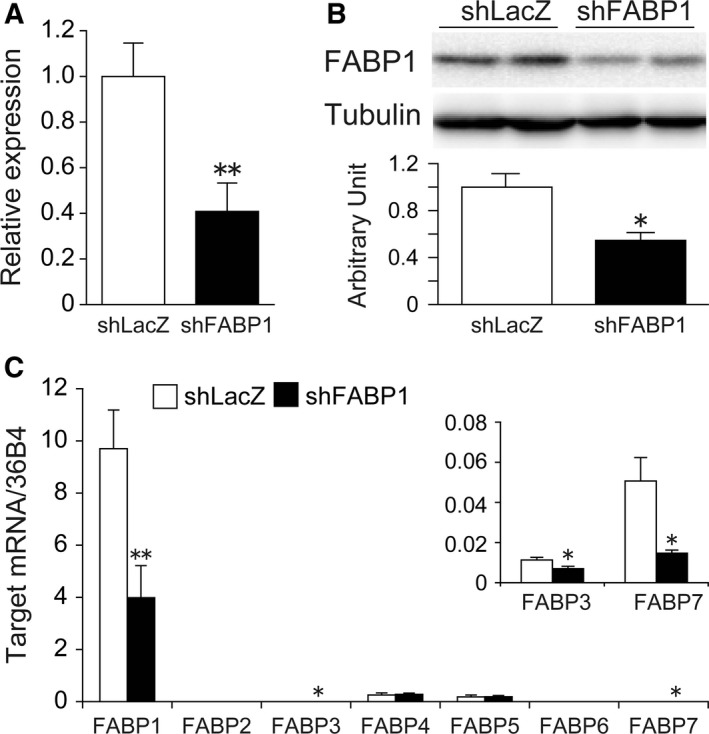
Adenovirus‐mediated FABP1 knockdown in HFD‐fed C57BL/6J mice. HFD‐fed C57BL/6J mice were injected with Ad‐shLacZ or Ad‐shFABP1 via the tail vein and killed 1 week later. (A) Real‐time PCR analysis of FABP1 expression in the liver. (B) Western blot analysis of FABP1 protein expression in the liver. The graph indicates the quantification of FABP1 protein. (C) mRNA expression of FABP family members was measured by real‐time PCR. Data are shown as the mean ± SEM (*n* = 7 per group). *P < 0.05, **P < 0.01.

To examine the effect of FABP1 knockdown on NAFLD, we generated adenovirus expressing shRNA for mouse FABP1 and injected it into HFD‐fed C57BL/6J mice via the tail vein. FABP1 mRNA expression in the liver of Ad‐shFABP1 injected mice was significantly reduced to approximately 40% of that in Ad‐shLacZ injected mice (Fig. 1A). The FABP1 protein expression in the liver of Ad‐shFABP1 mice was significantly decreased to 55% of that in Ad‐shLacZ‐injected mice (Fig. 1B). To investigate the compensation effects due to FABP1 knockdown, we measured the expression of other members of the FABP family. No difference in the mRNA expression of FABP2, 4, 5, or 6 in the liver was observed between Ad‐shFABP1‐injected mice and Ad‐shLacZ‐injected mice (Fig. 1C). Although we found significant decreases in FABP3 and FABP7 mRNA expression in Ad‐shFABP1‐injected mice, the expression of FABP3 and FABP7 in the liver was about 200–1000 times lower than that of FABP1 (Fig. 1C). The expression levels of FABP2 and 6 in the liver were four orders of magnitude lower than that of FABP1.

### Knockdown of FABP1 expression decreased liver weight and hepatic TG content

**Figure 2:**
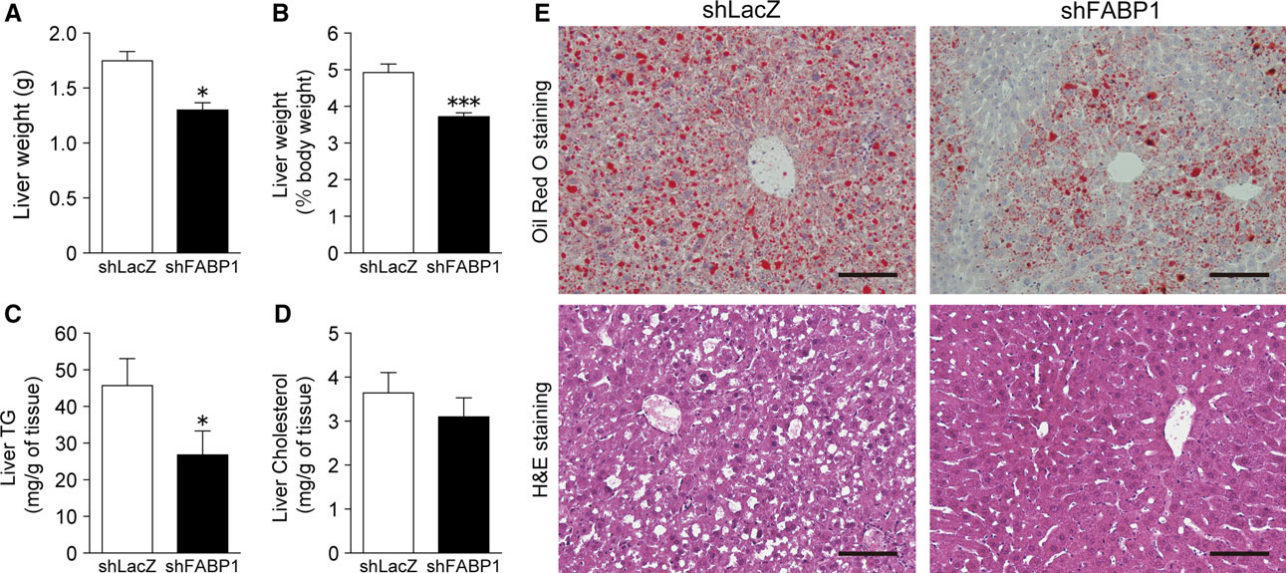
Knockdown of FABP1 expression decreased liver weight and hepatic TG content. HFD‐fed C57BL/6J mice were injected with Ad‐shLacZ or Ad‐shFABP1 via the tail vein (*n* = 7 per group). Mice were killed 1 week post injection. Liver weight (A, B), hepatic TG (C), and hepatic cholesterol (D) were determined. Data are shown as the mean ± SEM. *P < 0.05, ***P < 0.001. (E) Oil Red O staining and H&E staining of the liver in mice treated with Ad‐shLacZ or Ad‐shFABP1. Scale bar, 100 μm.

We investigated the effects of FABP1 knockdown on liver lipid accumulation. The liver weight in Ad‐shFABP1‐treated mice was significantly decreased by 20% compared with the mice injected with Ad‐shLacZ (Fig. 2A,B). The lipid content in the liver is shown in Fig. 2C,D. The TG concentration in the liver of Ad‐shFABP1‐injected mice was significantly lower than that in Ad‐shLacZ‐injected mice. The level of cholesterol in the liver of Ad‐shFABP1‐injected mice was comparable to the level observed in Ad‐shLacZ‐injected mice. The reduced TG content was confirmed by Oil Red O staining (Fig. 2E). No significant differences in serum TG level (Ad‐shLacZ: 153.9 ± 16.3 mg·dL^−1^; Ad‐shFABP1: 143.2 ± 34.6 mg·dL^−1^, *P* = 0.24) and serum cholesterol level (Ad‐shLacZ: 97.1 ± 17.0 mg·dL^−1^; Ad‐shFABP1: 78.3 ± 17.9 mg·dL^−1^, *P* = 0.09) were observed. In addition, FABP1 knockdown had no effect on body weight and food intake.

### FABP1 knockdown attenuated hepatic fatty acid and TG synthesis

**Figure 3:**
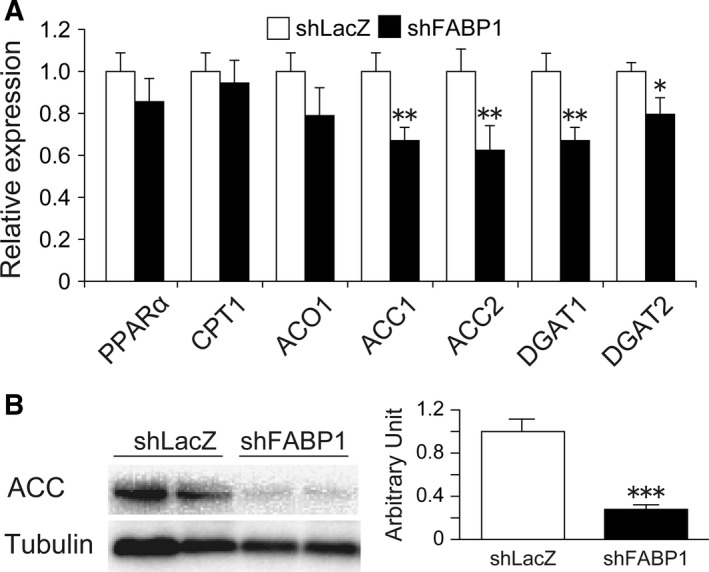
FABP1 knockdown attenuated hepatic fatty acid and TG synthesis. (A) Real‐time PCR analysis of genes involved in fatty acid oxidation and lipid synthesis in the liver of HFD‐fed C57BL/6J mice injected with Ad‐shLacZ or Ad‐shFABP1. (B) Western blot analysis of ACC protein expression in the liver. The graph indicates the quantification of ACC protein. Data are presented as the mean ± SEM (*n* = 7 per group). *P < 0.05, **P < 0.01, ***P < 0.001.

To understand the putative mechanisms involved in the improvement in fatty liver, we measured the expression of genes involved in fatty acid oxidation (Fig. 3A). The expression of peroxisome proliferator‐activated receptor α (PPARα) which regulates fatty acid oxidation was unchanged in the two groups. Significant differences in the mRNA expression of carnitine palmitoyltransferase 1 (CPT1) and acyl‐CoA oxidase 1 (ACO1), which are the rate‐limiting enzymes of beta‐oxidation in mitochondria and peroxisomes, respectively, were not observed. Next, we examined the expression of genes related to lipid biosynthesis [ACC and diacylglycerol acyltransferase (DGAT)] in the liver. The mRNA expression of ACC1 and ACC2 in the liver of Ad‐shFAPB1‐injected mice was significantly decreased compared with the mice injected with Ad‐shLacZ. The ACC protein expression in the liver of Ad‐shFABP1 mice was significantly decreased to 25% of that in Ad‐shLacZ‐injected mice (Fig. 3B). In addition, DGAT1 and DGAT2 gene expression in Ad‐shFABP1‐treated mice were also significantly decreased compared with Ad‐shLacZ‐treated mice. These data suggest that one of the reasons for the improvement in hepatic TG accumulation in Ad‐shFABP1‐treated mice was decreased *de novo* lipogenesis.

### FABP1 knockdown suppressed hepatic inflammation and oxidative stress

**Figure 4:**
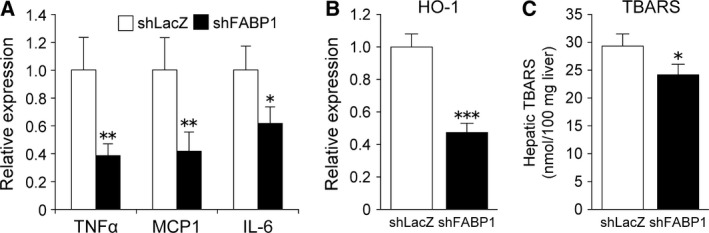
FABP1 knockdown suppressed hepatic inflammation and oxidative stress. Real‐time PCR analysis of genes related to inflammation (A) and oxidative stress (B) in the liver of HFD‐fed C57BL/6J mice injected with Ad‐shLacZ or Ad‐shFABP1. (C) Quantification of TBARS in the liver. Data are expressed as the mean ± SEM (*n* = 7 per group). *P < 0.05, **P < 0.01, ***P < 0.001.

Although the precise mechanism underlying the pathogenesis of NASH is not yet clear, inflammation and/or oxidative stress which are caused by lipotoxicity are thought to play important roles in the progression of NAFLD to NASH 22, 23. Accordingly, we assessed the impact of FABP1 silencing on the gene expression of cytokines and chemokines involved in hepatic inflammation. Ad‐shFABP1 significantly decreased the mRNA expression levels of monocyte chemotactic protein 1 (MCP1), tumor necrosis factor alpha (TNFα), and interleukin‐6 (IL‐6) in mouse liver (Fig. 4A). We also measured the gene expression of heme oxygenase‐1 (HO‐1), which is an important marker of oxidative stress, and found that the expression of HO‐1 gene in the liver of Ad‐shFABP1‐injected mice was significantly lower than that in Ad‐shLacZ‐treated mice (Fig. 4B). The levels of thiobarbituric acid‐reactive substances (TBARS), a marker of lipid peroxidation, were significantly reduced in the liver of Ad‐shFABP1‐treated mice (Fig. 4C). These results suggest that a reduction in FABP1 attenuated both inflammation and oxidative stress in the liver.

## Discussion

In this study, using an adenovirus‐mediated knockdown system, we demonstrated the effect of suppression of FABP1 function on NAFLD. We found that FABP1 knockdown in the liver decreased hepatic TG accumulation and improved hepatic inflammation and oxidative stress.

In order to decrease FABP1 function in the liver, we carried out adenovirus‐mediated knockdown of FABP1. As expected, the adenovirus expressing shRNA for FABP1 effectively suppressed the expression of FABP1 in the liver at both the mRNA and protein level. In studies using knockout mice, the effects of gene ablation are often compensated by the expression of other genes and this compensatory effect often eliminates or alters the results of gene ablation. For example, the loss of FABP4 was significantly compensated by increased expression of FABP5, and this compensation concealed the effects of FABP4 deficiency 24, 25. In addition, FABP5 gene ablation was compensated by an overexpression of FABP3 26. In this study, we observed a significant decrease in FABP3 and FABP7 mRNA expression in the liver of Ad‐shFABP1‐treated mice. The decreased expressions of FABP3 and FABP7 in the liver of global FABP1 gene‐ablated mice have been reported by Newberry *et al*. and Ong *et al*., respectively 15, 27. Our result was consistent with their results. The physiological significance of decreased expression of FABP3 and 7 is not clear. However, considering the mRNA abundance of FABP3, FABP7, and other FABP members in the liver, the effects of FABP1 silencing were probably not compensated by other FABP family members.

FABP1 gene silencing led to a decrease in liver weight and hepatic TG content. The liver weight of FABP1 knockout mice fed a HFD is highly divergent among studies 28. Mclntosh *et al*. 29 reported the increased liver weight in female FABP1 knockout mice (C57BL/6NCr background). By contrast, Newberry *et al*. 30 reported that female FABP1 gene‐ablated mice (C57BL/6J background) showed decreased liver weight. Our result was consistent with a recent Gajda's report where male FABP1 gene‐ablated mice (C57BL/6J background) fed a HFD showed a decreased liver weight 20. Although the mechanistic basis for this phenotypic divergence has not been fully elucidated, the difference in background strain (C57BL/6NCr vs C57BL/6J) was considered as a key contributor to the phenotypic difference.

Recent studies indicate that hepatic TG accumulation may be a protective response against lipotoxicity 23. However, since the stored TG is still a source of lipotoxic mediators, regulation of TG accumulation is still important for improving NAFLD 31. Theoretically, the major causes of liver lipid accumulation are increased uptake of free fatty acids (FFAs) into the liver, impaired fatty acid beta–oxidation, and increased *de novo* lipogenesis in the liver 6, 32. In addition, a reduction in lipid clearance can also contribute to lipid accumulation in the liver 33. Many studies have shown that FABP1 overexpression enhances fatty acid uptake and FABP1 gene ablation decreases hepatic fatty acid uptake 11, 12, 34. Therefore, one of the reasons for the improvement in fatty liver in Ad‐shFABP1‐treated mice seems to be decreased fatty acid uptake. To our knowledge, hepatic gene expression data have not been reported in male FABP1 knockout mice that fed a HFD *ad libitum*. However, male FABP1 null mice *pair‐fed* a HFD showed no significant change in the mRNA expression of CPT1 and ACO1 35. Consistent with this result, FABP1 repression did not affect the expression of genes involved in fatty acid oxidation. On the other hand, FABP1 reduction significantly decreased the expression of ACC and DGAT, key rate‐limiting enzymes in fatty acid biosynthesis, and TG formation, respectively 36, 37. Therefore, decreased *de novo* lipogenesis is probably involved in the amelioration of hepatic steatosis. Moreover, the activation of ACC2 is known to inhibit CPT1 activity via the production of malonyl‐CoA, a physiological inhibitor of CPT1 38, 39. Therefore, ACC plays an important role in regulating fatty acid oxidation as well as fatty acid biosynthesis. The decreased expression of ACC is known to decrease lipid synthesis and stimulate lipid oxidation, thereby improve hepatic steatosis 31. In our study, FABP1 suppression significantly decreased hepatic ACC expression at both mRNA and protein levels (Fig. 3A,B). Thus, although the expression level of CPT1 gene was not changed significantly, CPT1 enzyme activity may be elevated due to the decreased amount of malonyl‐CoA, resulting in enhanced fatty acid beta‐oxidation. These results suggest that in addition to reduced fatty acid uptake, decreased *de novo* lipogenesis and increased fatty acid beta‐oxidation may also contribute to the amelioration of fatty liver in mice treated with Ad‐shFABP1.

The pathogenesis of NASH has not been fully understood. However, a variety of factors such as oxidative stress, endoplasmic reticulum (ER) stress, FFAs, inflammatory cytokines, and gut‐derived endotoxins have been found to be involved in the progression of NASH 40. Hepatic oxidative stress plays a key role in NAFLD/NASH development. FABP1 is thought to serve as an endogenous cellular protectant and protects cells from the toxic effects of lipids. By binding potentially toxic ligands such as FFAs and heme, FABP1 attenuates the detergent effect of FFAs and the generation of reactive oxygen species by heme 14. Moreover, different to other FABP family members, FABP1 exerts scavenging effects through redox cycling of its methionine and sulfoxide reductase, thereby protecting cells from oxidative stress 41, 42. Therefore, we were concerned that the suppression of FABP1 expression would enhance the inflammatory response and oxidative stress in the liver. Surprisingly, the expression of inflammatory and oxidative stress markers was significantly decreased in the liver of Ad‐shFABP1‐treated mice compared with Ad‐shLacZ‐treated mice. FABP1 reduction also reduced hepatic lipid peroxidation. It was thought that although the decreased expression of FABP1 reduced the intracellular lipid‐binding capacity, the decreased fatty acid uptake and *de novo* lipogenesis led to decreased lipotoxicity, thereby lowering inflammation and oxidative stress in the liver.

In this study, we examined the potential of FABP1 suppression on the amelioration of NAFLD and found that FABP1 suppression exerted ideal effects for the treatment of NAFLD. As FABP1 plays important roles in the liver, complete inhibition of FABP1 may be harmful to the liver and the entire body. However, moderate inhibition of FABP1 function is considered to be a very promising and effective option for the treatment of NAFLD.

## Author contributions

HY and TK conceived and designed the experiments. TM, ME, and TK performed the experiments. TT and TK performed the histological analysis. TM and TK wrote the manuscript.

## Supporting information


**Table S1.** Real‐time PCR primer sequences.
