# Supplementary figures and images for: Silencing of FABP1 ameliorates hepatic steatosis, inflammation, and oxidative stress in mice with nonalcoholic fatty liver disease
Source: FEBS Open Bio. 2017 Jun 5;7(7):1009–16. doi: 10.1002/2211-5463.12240 (PMC5494302; doi:10.1002/2211-5463.12240)

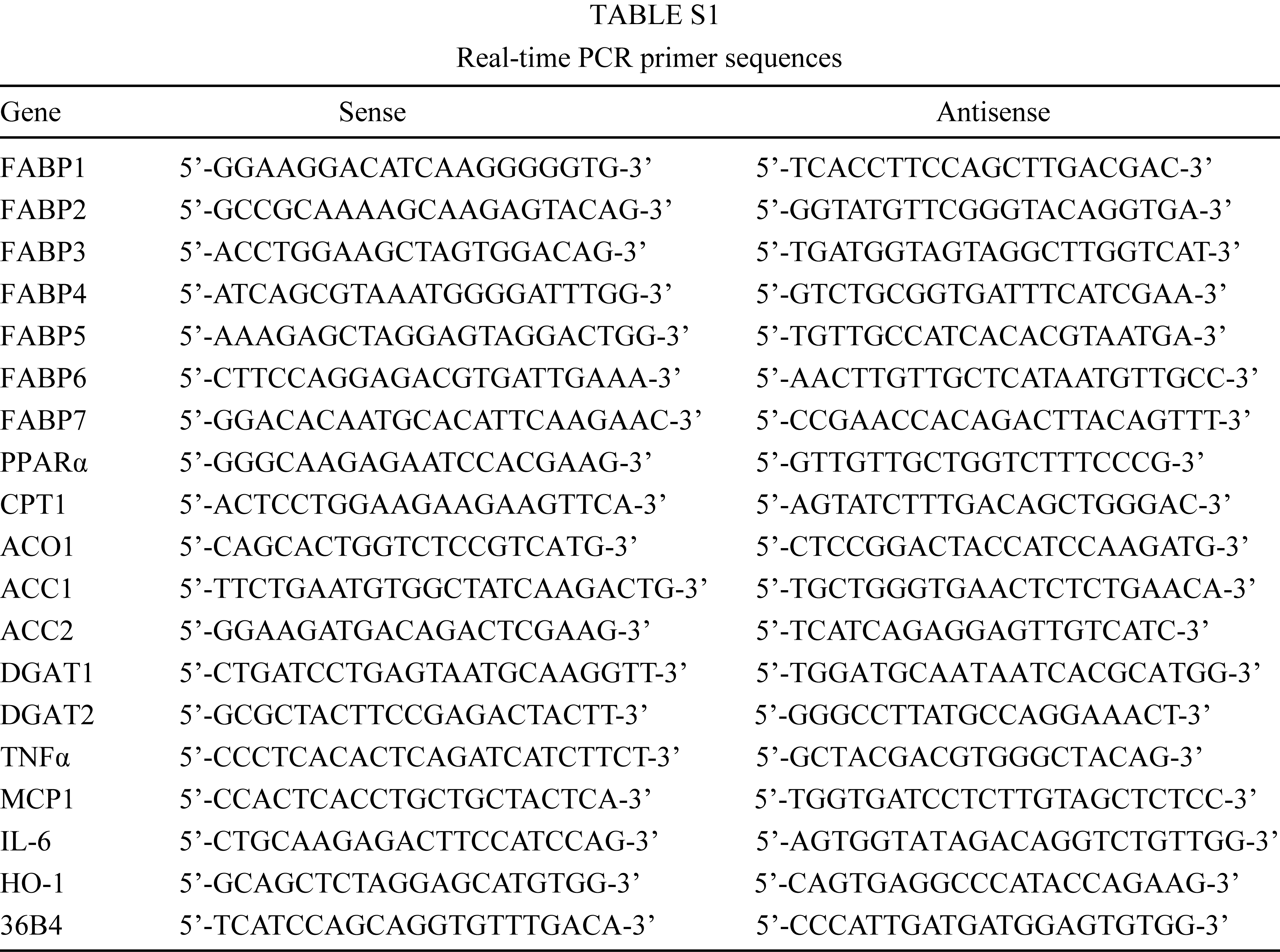

Supplement: Supplementary file 1 — Table S1. Real‐time PCR primer sequences. [file FEB4-7-1009-s001.tif]
